# Spatio-Temporal Distribution of Brucellosis in European Terrestrial and Marine Wildlife Species and Its Regional Implications

**DOI:** 10.3390/microorganisms10101970

**Published:** 2022-10-05

**Authors:** Tariq Jamil, Kadir Akar, Sevil Erdenlig, Jayaseelan Murugaiyan, Vassilios Sandalakis, Evridiki Boukouvala, Anna Psaroulaki, Falk Melzer, Heinrich Neubauer, Gamal Wareth

**Affiliations:** 1 Institute of Bacterial Infections and Zoonoses, Fredrich-Loeffler-Institut, 07743 Jena, Germany; 2 NRL for Brucellosis, Pendik Veterinary Control Institute, 34890 Istanbul, Turkey; 3 Faculty of Veterinary Medicine, Harran University, 63300 Şanlıurfa, Turkey; 4 Department of Biological Sciences, SRM University AP, Mangalagiri 522240, India; 5 Department of Clinical Microbiology and Microbial Pathogenesis, School of Medicine, University of Crete, 71110 Heraklion, Greece; 6 Hellenic Agricultural Organization—DIMITRA, Veterinary Research Institute, 57001 Thessaloniki, Greece

**Keywords:** terrestrial wildlife, marine wildlife, brucellosis, Europe

## Abstract

Brucellosis is an important bacterial zoonosis of domestic and wildlife species. This disease has a significant public health concern and is characterized by reproductive failure resulting in economic losses in the livestock industry. Among thirteen known species, *B. abortus, B. melitensis, B. suis,* and *B. canis* are human pathogens. Brucellosis has been extensively investigated in humans and domestic animals. However, the situation in wildlife is still not completely reported and studied. Therefore, a systematic literature search and screening were done to clarify the situation of brucellosis in wildlife in Europe. Sixty-five articles from a total of 13,424 reports published between 1991 and 2021 were selected, applying defined inclusion criteria. Wild boars and brown hares were the most often studied terrestrial wildlife species, whereas seals and porpoises were the most often investigated marine wildlife. Poland, Croatia, and Belgium showed the highest seroprevalences of wild boars caused by *B. suis* biovar 2. In marine wildlife, brucellosis was mainly caused by *B. ceti* and *B. pinnipedialis*. Most samples were from carcasses. Thus, sera could not be collected. It is worrisome that *B.* *abortus* and *B. melitensis* were reported from both terrestrial and marine wild animals, posing a zoonotic threat to people exposed to wild animals. Currently, there is no approved vaccine available for wild animals. The main challenges are the development of specific diagnostics and their validation for use in wildlife.

## 1. Introduction

Brucellosis is a major zoonotic infection of domestic animals and wildlife species worldwide [1,2]. Currently, there are 13 valid species of the genus *Brucella* (*B.*), including *B. abortus*, *B. melitensis*, *B. suis*, *B. canis*, *B. ovis, B. neotomae,* the “marine” *B. pinnipedialis, B. ceti* [3], *B. inopinata* [4], *B. microti* [5], *B. papionis* [6], *B. vulpis* [7], and the recently described *B. pseudogrignonensis* [8]. All these species are closely related, and the genus *Brucella* itself is closely related to *Ochrobactrum* [9], leading to the proposal to rename *Ochrobactrum*. This could result in 38 *Brucella* species. The disease has significant public health and economic impacts, particularly in middle- and low-income countries [10]. Brucellosis is mainly associated with reproduction failure in livestock and nonspecific symptoms in humans. Transmission occurs mainly via direct routes, i.e., contact with infected animals, or indirectly via contaminated fomites [11] and the consumption of contaminated unpasteurized dairy milk and products [12]. Despite the fact that brucellosis is a potential biological agent and occupational health hazard, treating the disease in farm animals is of limited practice due to the chronic nature of the disease. Resistance development in human isolates is still not well investigated [13]. A safe vaccine for use in humans is not available [14]. Human brucellosis is mainly reported in Latin America, the Middle East, Central Asia, and Mongolia [15,16], whereas it is sporadic in Europe and North America [17]. No reliable data are available for the African continent.

In the European Union (EU), livestock brucellosis caused by *B. abortus*, *B. melitensis*, and *B. suis* has been eradicated in farm animals in many countries [18]. In Croatia and Spain, eradication is nearly achieved, whereas in Greece, Italy, and Portugal, brucellosis remains a veterinary and public health concern with declining incidence rates [19,20]. In 2020, only six infected herds (extremely low prevalence (<0.001)) were reported in the officially brucellosis-free regions of the EU [19]. However, 0.38% (603/157,000) of bovine herds and 0.22% (349/160,000) of small ruminant herds still tested positive in brucellosis-affected regions of the EU (the lowest annual count since 2012) [19]. Overall, livestock brucellosis remained a rare event. On the other hand, canine brucellosis caused by *B. canis* showed an increasing trend in detectable cases, especially in Italy and the United Kingdom [19,21]. In contrast, human cases occurred due to infection transmission from wild animals and in travelers returning from disease-endemic areas after exposure. A total of 128 confirmed human cases were reported in 2020, with a decreasing trend since 2016 [19]. The notification rate was 0.03 cases per 100,000 people. In cases in which speciation could be confirmed, *B. melitensis* was the primary etiology in hospitalized patients, followed by *B. suis* [19]. Notifying livestock and human brucellosis is mandatory in at least 25 European countries, whereas a number of other countries have different/unspecified surveillance systems. Despite the efforts and money that have been spent, the infection persists in livestock and pet animals, and consequently, transmission to human occurs. On the other hand, the role of wildlife species is of great importance, but is often largely neglected. Brucellosis in wildlife has been a topic of interest for the past few decades since brucellae have been isolated in various terrestrial (e.g., wild boars, ruminants, canines, rodents, and reptiles) and marine (e.g., dolphins, whales, seals, and porpoises) animals, which possibly serve as reservoirs of brucellosis; spillover infections to domestic animals and humans [22,23]. Hence, wildlife brucellosis is not mandatory at all and data are scarce. 

This review aimed to get insights into the occurrence and epidemiological situation of brucellosis in European wildlife species.

## 2. Materials and Methods

### 2.1. Literature Source and Search Strategy

**Figure 1:**
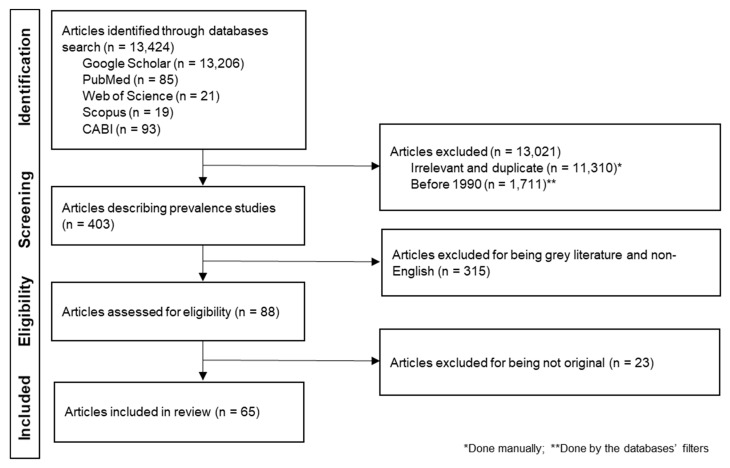
Flow diagram of the literature source and search strategy.

An initial literature search was conducted online by using the search words “brucellosis, *Brucella*, wild,” on Google Scholar (Google LLC, Mountain View, CA, USA), PubMed, Web of Science, Scopus, and the Centre for Agriculture and Bioscience International (CABI) (Wallingford, UK) search bar from November to December 2021. The countries’ names comprised fifty sovereign states in Europe. These countries were grouped into Eastern, South-Eastern, Central, Northern, Southern, and Western Europe. Dependent and extra-continental territories were not included except for Greenland, which was included in the literature search criteria based on socio-political context. Duplicates, conference abstracts and proceedings, reviews, and non-English articles were excluded. Only peer-reviewed original articles published between January 1991 and December 2021 were selected and analyzed (Figure 1).

### 2.2. Data Acquisition and Analysis

**Figure 2:**
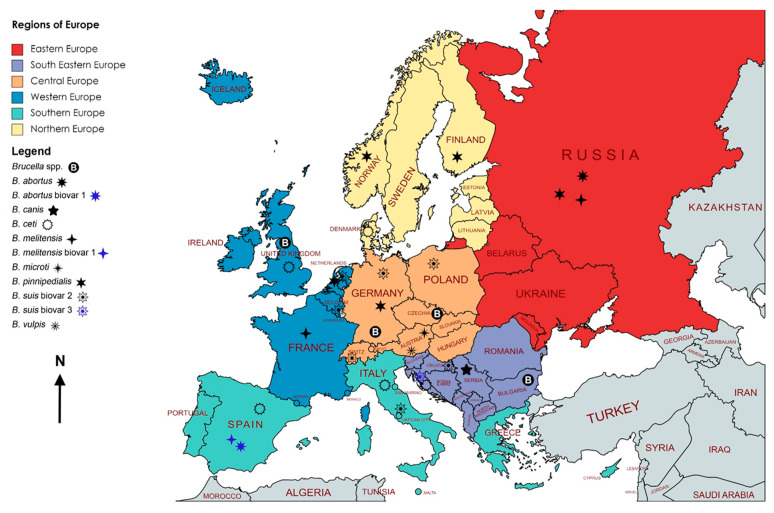
Map showing *Brucella* species and biovars isolated from European wildlife.

**Figure 3:**
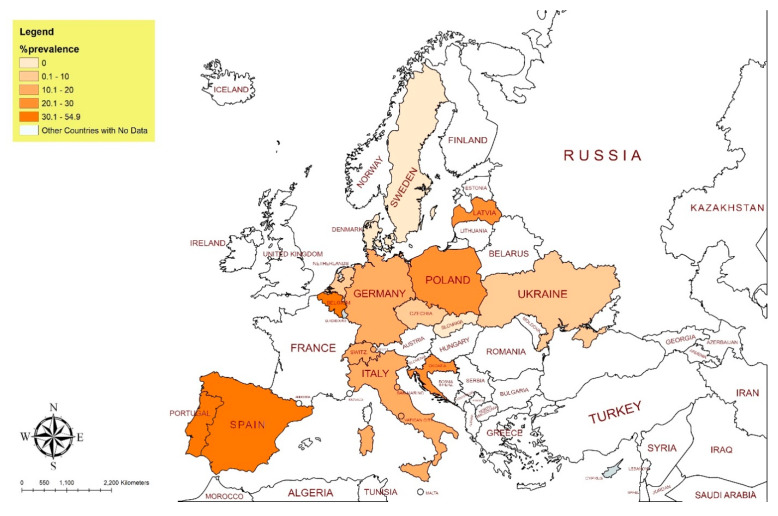
A map showing the prevalence of brucellosis in wild boars in Europe.

In total, 13,424 records were scrutinized (Google Scholar (*n* = 13,206); PubMed (*n* = 85); Web of Science (*n* = 21); Scopus (*n* = 19); CABI (*n* = 93)) for being relevant, original, full-length, and written in English language. Four hundred and three (403) articles were selected and screened. In total, sixty-five articles were found to be qualified and were included in this review (Figure 1). Information regarding geographical areas, host species, and seroprevalence reported was extracted, analyzed, and presented in Table 1 and Table 2. To have a spatial idea of the presence of *Brucella* spp. and the seroprevalence in Europe, maps were generated using open-source MapChart (https://www.mapchart.net/europe.html) (Figure 2 and Figure 3).

## 3. Results

### 3.1. Data Analysis

The data represented a total of 65 wildlife brucellosis reports from 25 European countries (42 reports from 20 countries for terrestrial wildlife and 23 reports from 11 countries for marine wildlife). Countries with only terrestrial wildlife brucellosis reports are Poland (*n* = 5), Czech Republic (*n* = 5), Austria (*n* = 3), Switzerland (*n* = 3), France (*n* = 2), and one report each from Serbia, Bulgaria, Slovenia, Ukraine, Denmark, Latvia, Iberian Peninsula (Spain and Portugal), Belgium, and Greenland. Countries reporting brucellosis in both terrestrial and marine wildlife are Germany (four in terrestrial and two in marine), Croatia (two in terrestrial and one in marine), Norway (one in terrestrial and three in marine), Sweden (one each in terrestrial and marine), Italy (seven in terrestrial and one in marine), and the Netherlands (one in terrestrial and two in marine). Russia, Finland, Spain, Iceland and the UK showed brucellosis only in marine wildlife, consisting of two, one, two, one and eight reports, respectively. It is worth mentioning that the article of Winkelmayer et al., 2005 [24], investigated brucellosis in the European hare at the Austrian–Czech border region and contains results for both countries. Therefore, this article is included twice—in the row of Austria and also in the row of the Czech Republic in Table 1. In the same context, in reporting marine wildlife brucellosis in the 10 aforementioned countries, the article of Sonne et al., 2018 [25], includes results of two countries, i.e., Norway and Sweden. Therefore, this study was included in rows of both countries in Table 2.

Most reports detected anti-*Brucella* antibodies by serology, e.g., the Rose Bengal test (RBT) and enzyme-linked immunosorbent assay (ELISA), especially in living terrestrial and marine animals. In contrast, isolation was a preferred choice in tissues from dead animals. Most isolation reports confirmed brucellosis only at the genus level, and species and subspecies levels were confirmed/typed only in recent reports. *B. suis* biovar (bv) 2 was the main finding in wild pigs. *B. abortus* bv 1 and *B. melitensis* bv 1 were isolated from red deer and Iberian wild goats described in one report from the Iberian Peninsula [26], while *B. melitensis* was isolated from Alpine ibex noted in two reports from France [27,28]. In one report from Croatia, *B. suis* bv 3 was listed for wild boars [29], and the isolation of *B. canis* was described for golden jackals in one report from Serbia [30]. In marine mammals, *B. pinnipedialis* was the main species found in seals, and *B. ceti* in dolphins and whales. The highest number of reports were published in 2018 (nine), followed by 2017 (seven), 2007 (five), 2014, 2015, and 2009 (four each), and 2021 (three).

### 3.2. Spatio-Temporal Distribution of Brucellosis in European Terrestrial Wildlife

#### 3.2.1. Eastern and South-Eastern Europe

Four countries, i.e., Russia, Belarus, Ukraine, and Moldova, were grouped into Eastern Europe in the study (Figure 2). Only one report from Ukraine was found describing 4.98% seroprevalence in wild boars in 2019 [31]. No evidence was found for PCR-based detection. Eleven countries, i.e., Slovenia, Croatia, Albania, Bosnia and Herzegovina, Bulgaria, Cyprus, Kosovo, Montenegro, North Macedonia, Romania, and Serbia, were grouped as South-Eastern European countries (Figure 2). Five reports described brucellosis in wild boars, golden jackals, and wild birds in this region. Wild boars showed the highest seroprevalence of 28.03% in 2003 and 23.11% in 2009 in Croatia, where *B. suis* bv 2 and 3 were confirmed by PCR [29,32]. In Serbia and Bulgaria, *B. canis* and *Brucella* spp. were detected in golden jackals [30] and wild birds [33], respectively, by PCR, while serology was not carried out. No evidence of anti-*Brucella* antibodies was found, and no amplifiable *Brucella* DNA was detected in wild boars of Slovenia in 2006 [34]. The prevalence and distribution of brucellosis in wild boars in Europe are shown in Figure 3.

#### 3.2.2. Central Europe

A total of eight countries comprising Poland, Slovenia, Czech Republic, Hungary, Austria, Switzerland, Liechtenstein, and Germany were grouped as central Europe (Figure 2). No reports were found for Slovenia, Liechtenstein, and Hungary. Again, wild boars were the most tested animals in this region, contributing to more than 75% of the animals tested. The highest seroprevalences reported came from Switzerland, i.e., 35.83% in 2011 [35], and was 9.8% in two rounds and 11.05% by ELISA in two studies in 2007 [36,37]. In Poland, it was 24.44% in 2015 [38] and, in Germany, was 22.02% in 2005 [39] and 12.09% in 2006 [40]. According to the National Reference Laboratory for Animal Brucellosis in Germany, *B. suis* bv 2 has been isolated many times from wild boar and hares in Germany during the period under review (unpublished data). Czech wild boars showed seroprevalences of 6.25% in 1993 [41] and 8.7% in 2002 [42]. By PCR, *B. suis* bv 2 was confirmed in Poland [43] and Switzerland [37] (Figure 3).

The brown hare was the second most studied wild animal in the region, constituting 14% of tested animals. The highest seroprevalence (3.54%) was found in Austria in 2005 [24]. In the Czech Republic, zero prevalence was reported in two studies in 1993 [41] and 2005 [24], and 1.62% seroprevalence was reported in 2007 [44]. In Germany, seroprevalence was reported at 0% in 2003 [45]. No serological report existed for Poland; however, *B. suis* bv 2 was confirmed by molecular detection in 2013 [43]. 

Other significantly tested terrestrial wildlife species were wild rodents, e.g., mice, common voles, and shrews, which showed a seroprevalence of 17.05%, 15.25%, and 7.96%, respectively, in 2017 [46]. *Brucella* spp. was confirmed by PCR in Czech voles and German shrews in 2007 [47] and 2017 [46], respectively. Various reports existed for wild deer and European bison populations in Poland [43,48,49,50,51] and the Czech Republic [41] but did not report detectable antibody levels and, hence, assumed zero seroprevalences in this wildlife. In Austria, *B. microti* and *B. vulpis* were identified by molecular testing in red foxes from mandibular lymph nodes in 2009 [52] and 2016 [7], respectively.

#### 3.2.3. Northern Europe

Norway, Sweden, Finland, Denmark, Estonia, Latvia, and Lithuania were grouped into Northern Europe (Figure 2). A total of three reports from Denmark, Latvia, and Sweden were found for wild boars, as well as one report from Norway on samples collected from reindeer. A seroprevalence of 22.51% was reported in Latvian wild boars in 2018, where *B. suis* bv 2 was identified by culture and PCR [53]. No antibodies were detected in Swedish and Danish wild boars in 2018 [54] and 2020 [55], respectively, and the same finding was reported for Norwegian reindeer in 1999 [56].

#### 3.2.4. Southern Europe

Seven countries, including Portugal, Spain, Italy, San Marino, Vatican City, Malta, and Greece, were grouped as Southern Europe, out of which three had reports of brucellosis in terrestrial wildlife (Figure 2). A seroprevalence of 33% was found in Iberian (Spain and Portugal) wild boars, where *B. suis* bv 2 was detected in all seropositive animals by isolation and molecular identification for ten years (1999–2009) [26]. Barbary sheep, mouflons, roe deer, and fallow deer did not show seropositivity. However, chamois and red deer showed 0.78% and 0.4% seropositivity, respectively. In addition, *B. melitensis* bv 1 and *B. abortus* bv 1 were found in one Iberian wild goat and one red deer, respectively [26]. 

In Italy, 19.76% seroprevalence was reported in 2,267 wild boars sampled between 2001–2007 [57]. All seropositive boars yielded *B. suis* bv 2 in culture. A 6.1% seroprevalence was reported without confirming the etiology in 2015 [58]. *B. suis* bv 2 was confirmed in 2017 [59]. A seroprevalence of 13.5% was found without confirming the etiology in 2020 [60]. *B. suis* bv 2 was confirmed recently with a seroprevalence of 5.74% in wild boars in 2021 [61]. Di Francesco et al. (2015) reported a 9.09% seroprevalence in Marsican brown bears in 2015 [62]. No detectable *Brucella* DNA was reported in wild birds’ fecal samples in 2021 [63]. No anti-*Brucella* antibodies were found in 236 chamois and 207 roe deer sampled in Italy between 1998–2001 [64].

#### 3.2.5. Western Europe

Nine countries, including the United Kingdom, Ireland, Iceland, the Netherlands, Belgium, Luxembourg, Monaco, Andorra, and France, were grouped into Western Europe (Figure 2). In Belgium, an apparent seroprevalence of 54.88% was reported in 1168 wild boars sampled between 2003–2007 [65]. In the Netherlands, 6.36% seroprevalence was reported in 2057 wild boars sampled between 2010–2015 [66]. Both studies confirmed *B. suis* bv 2 by culture and molecular typing. In France, anti-*Brucella* antibodies were detected in red deer, chamois, and alpine ibex [27], and *B. melitensis* was confirmed in Alpine ibex [28].

#### 3.2.6. Special Territories 

Greenland was grouped as a special territory. A seroprevalence of 6.25% was reported in 96 polar bears, and 0% was reported in 32 muskoxen examined by serology and without confirmation by culture examination in 2018 [67].

**Table 1:** Brucellosis in European terrestrial wildlife.

No.	Countries	Region	Host	Serology			Molecular Identification	Citation
				No. Tested	No. Positive	%Prev.		
1	Ukraine	Eastern Europe	Wild boars	1344	67	4.98		[31]
2	Croatia	South-Eastern Europe	Wild boars	264	74	28.03	*B. suis* bv 2	[32]
				424	98	23.11	*B. suis* bv 2 and bv 3	[29]
3	Serbia		Golden Jackals	216	--	--	*B. canis*	[30]
4	Bulgaria		Wild birds	706	--	--	*Brucella* spp.	[33]
5	Slovenia		Wild boars	178	0	0		[34]
6	Austria	Central Europe	Red foxes				*B. microti*	[52]
							*B. vulpis*	[7]
			Brown hare	311	11	3.54		[24]
7	Czech Republic		Wild boars	204	18	8.7		[42]
				32	2	6.25		[41]
			Brown hare	73	0	0		[24]
				1051	17	1.62		[44]
				23	0	0		[41]
			Roe deer	33	0	0		[41]
			Red deer	24	0	0		
			Fallow deer	4	0	0		
			Mouflon	2	0	0		
			Common vole	4	--	--	*Brucella* spp.	[47]
8	Germany		Wild boars	763	168	22.02		[39]
				885	107	12.09		[40]
			Brown hare	321	0	0		[45]
			Shrews	113	9	7.96	*Brucella* spp.	[46]
			Voles	295	45	15.25		
			Mouse	129	22	17.05		
9	Poland		European bison	60	0	0		[48]
				122	0	0		[49]
				240	0	0		[51]
			Deer	183	--	--	No isolate was achieved	[50]
			Wild boars	235	--	--	*B. suis* bv 2	
				4407	1077	24.44		[38]
10	Switzerland		Wild boars	810	90	11.05	No isolate was achieved	[36]
				* 611	27	4.42	*B. suis* bv 2	[37]
				^+^ 1215	153	12.59		
				^±^ 462	66	14.28		
				240	86	35.83		[35]
11	Denmark	Northern Europe	Wild boars	240	0	0		[55]
12	Latvia		Wild boars	1044	235	22.51	*B. suis* bv 2	[53]
13	Norway		Reindeer	5792	0	0		[56]
14	Sweden		Wild boars	286	0	0		[54]
15	Iberian Peninsula (Spain and Portugal)	Southern Europe	Barbary sheep	8	0	0		[26]
			Mouflon	75	0	0		
			Iberian wild goat	1086	1	0.09	*B. melitensis* bv 1	
			Chamois	1410	11	0.78		
			Roe deer	285	0	0		
			Fallow deer	342	0	0		
			Red deer	5821	19	≤0.4	*B. abortus* bv 1	
			Wild boars	4454	1470	33	*B. suis* bv 2	
16	Italy		Wild boars	570	35	6.1		[58]
			Wild boars	2267	448	19.76	*B. suis* bv 2	[57]
			Brown bears	22	2	9.09		[62]
			Wild boars	389	--	--	*B. suis* bv 2	[59]
			Wild boars	434	58	13.5		[60]
			Wild Birds	121				[63]
			Wild boars	287	16	5.74	*B. suis* bv 2	[61]
17	Belgium	Western Europe	Wild boars	1168	641	54.88	*B. suis* bv 2	[65]
18	France		Roe deer	44				[27]
			Red deer	30	1			
			Chamois	55	1		*B. melitensis*	
			Alpine ibex	24	12			
			Alpine ibex	339	88		*B. melitensis*	[28]
19	Netherlands		Wild boars	2057	131	6.36	*B. suis* bv 2	[66]
20	Greenland	Special territory	Polar bears	96	6	6.25		[67]
			Greenland muskoxen	32	0	0		

* First round; ^+^ Second round; ^±^ Included in both rounds; (--) = Not performed.

### 3.3. Spatio-Temporal Distribution of Brucellosis in European Marine Wildlife

#### 3.3.1. Eastern and South-Eastern Europe

On the Russian Bering Island, DNA sequences from rectal-swabs matched *B. abortus*, *B. melitensis,* and *B. pinnipedialis* in 3 of 78 Asian sea otters [68]. Another study reported seropositive samples in 5.63% of Caspian seals and 75% of Beluga whales, whereas seven Baikal seals and six Ringed seals were seronegative on Russian territory in 2018 [69]. In the Croatian Adriatic Sea, *B. ceti* ST27 was isolated and identified from one of five bottlenose dolphins in 2016 [70].

#### 3.3.2. Central Europe

Two studies were found for Germany. In the German North Sea in 2008, *Brucella* isolates were recovered from common seals (47/426), harbor porpoises (2/298), and grey seals (1/34). Based on PCR-restriction fragment length polymorphism (PCR-RFLP), 47 were classified as *B. pinnipedialis* and the other 2 as *B. ovis* based on the presence of the “*omp2b*” gene pattern [71]. In another study, 2105 harbor seals were sampled between 1996–2014, and 359 *Brucella* spp. isolates were recovered and 47 isolates were confirmed as *B. pinnipedialis* [72]. No studies on brucellosis in marine species were reported in other central European countries.

#### 3.3.3. Northern Europe

A total of nine seropositive animals were detected from 29 tested and apparently healthy Norwegian hooded seals. Eleven *B. pinnipedialis* isolates were obtained from seropositive animals and two seronegative animals. No isolates were found in ringed seals [73]. A seroprevalence of 15.57% was found in Norwegian hooded seals with one *B. pinnipedialis* isolate in 2013 [74]. No seropositive animals were detected in harp and hooded seals from Norway in 2018 [25]. The same study reported 2 out of 12 seropositive ringed seals in Sweden [25]. *B. pinnipedialis* was isolated from 3 out of 122 Baltic grey seals in Finnish samples from 2013 to 2015 [75]. 

#### 3.3.4. Southern Europe

On the Spanish Mediterranean coast, two out of sixteen striped and one out of two bottlenose dolphins were reported as seropositive. Four Risso’s dolphins, one short-beaked dolphin, and one fin whale tested seronegative in 2001 but the culture was not conducted [76]. However, three *B. ceti* isolates were reported, two from striped dolphins and one from a bottlenose dolphin (all were seropositive by RBT) on the Catalonian Mediterranean coast in 2014 [77]. In Italy, eight *B. ceti* isolates were reported from one seropositive and seven seronegative striped dolphins in 2020 [78].

#### 3.3.5. Western Europe

*B. ceti* ST23 was isolated and confirmed in 7 out of 112 harbor porpoises stranded on Dutch coasts between 2008 and 2011 [79]. *B. pinnipedialis* ST25 was isolated from 16 out of 40 seropositive harbor seals in 2018 [80]. A total of 49% (69/140) of harbor seals, 32% (10/31) of grey seals, 28% (5/18) of harbor porpoises, 0% (0/45) of Baikal seals, and one short-beaked common dolphin were reported seropositive by RBT in the North Sea in 1996, resulting in eight isolates of *Brucella* spp. from harbor porpoises, harbor seals, and the dolphin [81]. Atypical isolates of *Brucella* spp. were isolated from nine seals, eight cetaceans, and one otter [82]. Another study reported seropositivities of 9.68% (6/62) in grey seals, 8.33% (1/12) in common seals, 31.42% (11/35) in harbor porpoises, 31.03% (9/29) in common dolphins, one striped dolphin, one bottlenose dolphin, one killer whale, and one pilot whale on English and Welsh coasts without culture examination in 1997 [83]. Two *B. ceti* isolates were obtained from one long-finned pilot whale and one of ten Sowerby’s beaked whales found on Scottish coasts in 2015 [84]. 25.36% (87/343) harbor seals tested positive by ELISA between 1997–2012 [85]. *B. ceti* was also found in Risso’s dolphin and killer and minke whales in 2021 and 2017 [86,87]. A total of 1.85% harp seals, 35.04% hooded seals, 10.2% ringed seals, 11.11% fin whales, 14.28% sei whales and 7.87% mink whales were detected seropositive with isolation of *Brucella* spp. from a minke whale in North Atlantic Ocean [88].

**Table 2:** Brucellosis in European marine wildlife.

No.	Countries	Region	Host	Serology			Molecular Identification	Citation
				No. Tested	No. Positive	% Prevalence		
1	Russia	Eastern Europe	Sea otters	78	--	--	*B. abortus, B. melitensis* and *B. pinnipedialis*	[68]
			Caspian seals	71	4	5.63		[69]
			Baikal seals	7	0	0		
			Ringed seals	6	0	0		
			Beluga whales	4	3	75		
2	Croatia	South-Eastern Europe	Bottlenose dolphins	4	--	--	*B. ceti* ST27	[70]
3	Germany	Central Europe	Harbor seals	2105	--	--	*B. pinnipedialis*	[72]
			Common seals	426			*B. pinnipedialis*	[71]
			Harbor porpoises	298				
			Grey seals	34				
			Hooded seals	3				
			Common dolphins	3				
			White-beaked dolphin	1				
			Ringed seal	1				
			Pilot whale	1				
			Minke whale	1				
6	Finland	Northern Europe	Grey seals	122	--	--	*B. pinnipedialis*	[75]
5	Norway		Harp and hooded seals	9	0	0		[25]
			Hooded seals	379	59	15.57	*B. pinnipedialis*	[74]
			Hooded seals	29	9	31.03	*B. pinnipedialis*	[73]
			Ringed seals	20	0	0	No isolate was achieved	
6	Sweden		Ringed seals	12	2	16.67		[25]
			Harp seals	6	0	0		
			Hooded seals	3	0	0		
7	Spain	Southern Europe	Striped dolphins	2	2		*B. ceti*	[77]
			Bottlenose dolphin	1	1		*B. ceti*	
			Striped dolphins	16	2	12.5		[76]
			Risso’s dolphins	4	0	0		
			Bottlenose dolphins	2	1	50		
			Short-beaked common dolphin	1	0	0		
			Fin whale	1	0	0		
8	Italy		Striped dolphins	8	1	12.5	*B. ceti*	[78]
9	Netherlands	Western Europe	Wild grey seals	11	1	9.09		[80]
			Harbor seals	40	16	40	*B. pinnipedialis*	
			Porpoises	112	--	--	*B. ceti* ST23	[79]
10	UK		Common seals	140	69		*Brucella* spp.	[81]
			Harbor porpoise	18	5			
			Baikal seals	45	0			
			Grey seals	31	10			
			Common dolphin	1	1			
			Atlantic white-sided dolphin	1			*Brucella* spp.	[82]
			Striped dolphin	2				
			Hooded seal	1				
			Grey seal	1				
			European otter	1				
			Bottlenose dolphin	1			*Brucella* spp.	[89]
			Grey seal	62	6	9.68		[83]
			Common seal	12	1	8.33		
			Harbor porpoise	35	11	31.42		
			Common dolphin	29	9	31.03		
			Striped dolphin	4	1	25		
			White-beaked dolphin	4	0	0		
			Atlantic white-sided dolphin	2	0	0		
			Bottlenose dolphin	1	1			
			Pilot whale	1	1			
			Risso’s dolphin	1	0	0		
			Killer whale	1	1			
			Blainville’s beaked whale	1	0	0		
			Long-finned pilot whale	1			*B. ceti*	[84]
			Sowerby’s beaked whale	10			*B. ceti*	
			Harbor seals	343	87	25.36	*Brucella* spp.	[85]
			Risso’s dolphin	1	--	--	*B. ceti*	[86]
			Killer whale	1	--	--		
			Common minke whale	1			*B. ceti*	[87]
11	North Atlantic Ocean		Harp seals	811	15	1.85		[88]
			Hooded seals	137	48	35.04		
			Ringed seal	49	5	10.20		
			Bearded seal	16	0	0		
			Fin whale	108	12	11.11		
			Sei whale	49	7	14.28		
			Minke whale	216	17	7.87	*Brucella* spp.	

(--) = Not performed.

## 4. Discussion

Wildlife interacts with humans, domestic livestock, and pet animals and, thus, can act as reservoirs and sources for the spillover of several infections and zoonotic diseases to the human interface [90]. The potential role of wildlife as a source of human zoonoses is a significant public health concern [91]. The study of brucellosis in wildlife is neglected and has only recently received increasing attention. [92,93]. The impacts of infected wildlife on public health and domestic animals are spillover and reservoir (sustainability). In addition, human activities that put persons at risk, such as hunting, dressing, meat handling, consumption, wildlife sampling, and management in intensive settings, contribute to the transmission of disease [94]. Thus, the current review provides a comprehensive, evidence-based assessment of existing literature and available data on brucellosis in terrestrial and marine wildlife in Europe.

Striking seroprevalences of brucellosis were found in wild boars (Figure 3), but also in European brown hares, red foxes, and wild deer captured in Belgium, Poland, Croatia, the Iberian Peninsula, Italy, and Latvia. *B. suis* bv 2 was the main etiological agent of brucellosis in wild boars; however, it was also confirmed in brown hares in Poland in 2013 [43]. Although *B suis* bv 1 is causing severe infection in humans, infections with *B. suis* bv 2 results in a very mild course in humans. Biovar 1 may also cause infection in wild boars and hares with severe consequences for hunters and farmers if it is capable of becoming enzootic in free areas [95]. *B. suis* bv 2 may be transmitted to domesticated animals. It has been isolated from domestic pigs in Egypt [96], but it is rarely described as a cause of human brucellosis among hunters [97]. Nevertheless, the presence of *B. suis* bv 2 in wild boars and hares is a potential source of spillover and spill-back infection in wildlife and domestic livestock. *B. suis* bv 3 was confirmed in Croatian wild boars and *B. melitensis* bv 1 and *B. abortus* bv 1 were isolated from wild ruminants in the Iberian Peninsula, which pose a direct threat to human health. Wild bison (*Bison bison*) and elk (*Cervus canadensis*) in the American Greater Yellowstone Ecosystem [98], African buffalo (*Syncerus caffer*) in south-east Africa [93], and Alpine ibexes (*Capra ibex*) in the French Alps [28] are considered self-sustaining reservoirs of *B. abortus* and *B. melitensis*. In this scenario, reducing spill-back infection from wildlife to livestock and humans would be of greater importance [99]. For this purpose, vaccination, culling, and treatment of wild animals would be interesting but is highly questionable. Hence, French authorities decided to reduce the number of strictly protected ibex dramatically. The reintroduction of endangered bison in the EU and the use of free-ranging water buffaloes for landscape conservation need special caution and care from the veterinary public health service, therefore.

*B. canis* has caused canine brucellosis in wild canines, e.g., jackals [30] and pet dogs [21]. It is an emerging threat in Europe in the camel class and a threat to human and animal health; of special concern are large stray dog populations. Infected dogs act as transmitters between humans and domestic and wildlife populations, as confirmed previously in Egypt [100] and Pakistan [101]. To the best of our knowledge, no reports have been found for *B. ovis* in European terrestrial wildlife [99], except for a report from Germany, in which two *B. ovis* isolates were identified from marine mammals based on the presence of the “*omp2b*” gene pattern [71]. In marine wildlife, brucellosis causes abortions as well [102]. Most of the *Brucella* isolates identified were *B. pinnipedialis* followed by *B. ceti* from harbor porpoises, dolphins, and seals, but *B. abortus* and *B. melitensis* have been isolated from Russian sea otters. The latter findings indicate a potential zoonotic threat to humans and people consuming raw seafood [68,103].

As in domestic animals, serological tests remain the mainstay in diagnosing wildlife brucellosis, e.g., the Rose Bengal Test (RBT) and enzyme-linked immunosorbent assay (ELISA). The reason might be easy handling, availability, and extensive use in domestic animals. Moreover, the antigens used in wildlife testing are smooth lipopolysaccharides (LPS), which cross-react with antibodies provoked by *B. abortus*, *B. melitensis,* and *B. suis* [104]. Anti-smooth *Brucella* LPS antibodies indicate either an active infection or exposure to *Brucella* in the recent past. Unfortunately, these tests must be applied without validation due to missing numbers of positive (and negative) sera for the variety of wildlife species to be tested. Competitive ELISA or fluorescent polarization assays (FPA) may be used as they do not depend upon species-specific reagents [105,106]. However, standardization and validation of these diagnostic tests would still be needed [107].

Serology has many pitfalls. Antibody titers may decrease with time and pretend lower prevalence. Furthermore, the detection of latent infection by serology is hampered. Cross-reaction with the LPS of other Gram-negative pathogens reduces sensitivity. In this scenario, the predictive values of the test would be more favorable than the intrinsic values of the test. On the other hand, canine brucellosis cannot be detected with the smooth LPS antigens used for the diagnosis of livestock brucellosis and needs antigens prepared from the rough LPS of *B. canis* [101]. Cytoplasmic proteins, however, could be of diagnostic importance in this scenario [108,109].

Furthermore, the quality of the serum obtained can influence the selection of the diagnostic tests applied, e.g., strong anti-complementary activity in wild boar and canine sera due to the presence of hemolysis, or other reasons due to unconducive field conditions, amateur collectors, e.g., hunters, and the time-lapse from collection to the submission of the specimens may interfere with sensitivity and specificity. Hence, establishing a definitive brucellosis diagnostic criterion in wildlife valid for all cases is challenging. Nevertheless, the isolation of brucellae remains the gold standard in wildlife too. Brucellosis has been successfully eradicated in domestic animals in many EU countries by applying a test-and-slaughter policy (after banning vaccination) [20,110]. However, this approach is not acceptable for endangered wildlife. Vaccination can be recommended for reservoir species, but no vaccine is available for brucellosis in wildlife [104]. Additionally, ensuring a representative and achievable sampling frame in the wildlife population for diagnostic/epidemiological purposes always remain a problem. 

## 5. Conclusions

This manuscript is aimed at updating the knowledge of brucellosis in European wildlife. A large number of reports exist for wild boars, followed by brown hares, red foxes, and wild deer. There was no significant public health threat from wildlife brucellosis as most of the infections occurred due to *B. suis* bv 2, *B. ceti,* and *B. pinnipedialis*, which seem to not be of zoonotic importance. However, the presence of *B. suis* bv 3, *B. abortus*, *B. melitensis*, and *B. canis* pose a significant zoonotic threat. They were identified in terrestrial and marine wildlife. Thus, wildlife poses a spillover and spill-back infection threat, which needs to be controlled. Definitive diagnostic criteria, the collection of viable specimens, and establishing a representative sampling frame would be highly desirable to collect more accurate epidemiological information on the prevalence of wildlife brucellosis and its etiology. Vaccination of wild reservoir hosts could be sensible. For this, developing a safe vaccine would be fruitful. The culling of infected wildlife remains an ethical question to be answered carefully. Protection should be used while handling dead animals and awareness of foodborne infection should be raised among consumers.
